# Application of Reproductive Technologies to the Critically Endangered Baw Baw Frog, *Philoria frosti*

**DOI:** 10.3390/ani13132232

**Published:** 2023-07-07

**Authors:** Aimee J. Silla, Rebecca J. Hobbs, Deon J. Gilbert, Damian Goodall, Marissa L. Parrott, Adam Lee, Justine K. O’Brien, Phillip G. Byrne

**Affiliations:** 1 School of Earth, Atmospheric and Life Sciences, University of Wollongong, Wollongong, NSW 2522, Australia; 2 Taronga Institute of Science and Learning, Taronga Conservation Society Australia, Mosman, NSW 2088, Australia; 3 Wildlife Conservation and Science, Zoos Victoria, Elliott Avenue, Parkville, VIC 3052, Australia

**Keywords:** amphibian, captive breeding, reproductive technologies, ART, gamete-release, oviposition, spawning, sperm, spermiation, cryopreservation, biobanking

## Abstract

**Simple Summary:**

The integration of reproductive technologies into conservation breeding programs can contribute to the propagation and genetic management of threatened amphibian species. Here, we report on the first application of reproductive technologies to the critically endangered Baw Baw frog, *Philoria frosti*. We present the results of the successful use of protocols for hormone-induced spawning in male–female pairs, hormone-induced sperm-release in isolated males, and sperm cryopreservation.

**Abstract:**

Reproductive technologies (RTs) can assist integrated conservation breeding programs to attain propagation targets and manage genetic diversity more effectively. While the application of RTs to enhance the conservation management of threatened amphibians has lagged behind that of other taxonomic groups, a recent surge in research is narrowing the divide. The present study reports on the first application of RTs (hormone-induced spawning, hormone-induced sperm-release, and sperm cryopreservation) to the critically endangered Baw Baw frog, *Philoria frosti*. To determine the effect of hormone therapy on spawning success, male–female pairs were administered either 0 μg/g gonadotropin-releasing hormone agonist (GnRHa), 0.5 μg/g GnRHa, or 0.5 μg/g GnRHa + 10 μg/g metoclopramide (MET) (*n* = 6–7 pairs/treatment), and the number of pairs ovipositing, total eggs, and percent fertilisation success were quantified. To determine the effect of hormone therapy on sperm-release and to establish the peak time to collect sperm post-hormone administration, males were administered 0 IU/g (*n* = 4), or 20 IU/g hCG (*n* = 16). Total sperm, sperm concentration, and percent viability were quantified at 0, 2, 4, 6, 8, 10, and 12 h post-hormone administration. Overall, the percentage of pairs ovipositing was highest in the GnRHa + MET treatment, with 71% of pairs ovipositing, compared to 57% and 33% of pairs in the GnRHa and control treatments, respectively. The quantity of sperm released from males in response to hCG peaked at 4 h post-hormone administration, though it remained high up to 12 h. The percent sperm viability also peaked at 4 h post-administration (94.5%), exhibiting a steady decline thereafter, though viability remained above 77% throughout the 12 h collection period. The remaining sperm samples (*n* = 22) were cryopreserved using established protocols and biobanked for long-term storage and future conservation applications. The mean post-thaw sperm viability was 59%, and the percent total motility was 17%. The results from this preliminary study will direct further applications of RTs to the critically endangered Baw Baw frog to assist with species recovery.

## 1. Introduction

The primary objective of conservation breeding programs (CBPs) is to aid threatened species recovery by maintaining genetically representative captive assurance colonies ex situ, whilst contributing to active conservation management [1]. This objective may be achieved through a combination of actions, including generating viable offspring for the maintenance of captive colonies, conservation research, and release in situ to bolster populations (via population augmentation, translocation and reintroduction); population monitoring post-release, collection, and banking of recoverable genetic material; acting as emissaries for conservation education; and generating financial support for species recovery [1]. Reproductive technologies (including hormone therapies, sperm cryopreservation, and assisted fertilisation) can assist integrated CBPs to achieve propagation targets, manage genetic diversity, and safeguard genetic resources for future conservation applications [2,3]. While the development and application of reproductive technologies to species conservation has largely focused on mammalian taxa, a surge in research has led to an increase in reports of the successful application of technologies to a greater diversity of taxa, including threatened coral, fish, and amphibians [4].

The development and application of reproductive technologies to amphibians has increased substantially in recent decades following the formal recognition of the extent of amphibian declines and the release of the first Amphibian Conservation Action Plan (ACAP) in 2007 [5]. One of the main focal areas of research has been the development of hormone therapies, which have been successfully applied to a growing number of amphibian species to induce spawning in male–female pairs or groups, egg-release in isolated females, and sperm-release in isolated males (reviewed in [2,6,7,8,9,10]). The two most commonly employed exogenous hormones for reproductive hormone therapy in amphibians are synthetic gonadotropin-releasing hormone agonist (GnRHa) and purified human chorionic gonadotropin (hCG) [2,8]. In recent years, there has also been growing interest in the administration of GnRHa in combination with a dopamine antagonist (e.g., domperidone, pimozide, or metoclopramide (MET)), which is thought to block dopaminergic inhibition and enhance GnRH-stimulated luteinizing hormone (LH) release [6,11]. Importantly, while previous research provides a solid framework for the application of hormone therapies to novel amphibian species, protocols and expected outcomes exhibit a level of species specificity [2,10]. This is particularly true for the speed and length of time that male amphibians release sperm in response to hormone administration [2,10], which is imperative to quantify in order to maximise the quantity and quality of sperm collected for biobanking.

The critically endangered Baw Baw frog, *Philoria frosti*, was considered relatively abundant within its former range prior to 1984, after which time the species experienced dramatic decline and range restriction [12]. Current estimates indicate that there are less than 500 individuals remaining in the wild (D.J. Gilbert, pers. comm.). The onset and precipitous nature of the decline of this species is indicative of the amphibian chytrid fungus (*Batrachochytrium dendrobatidis, Bd*), being the primary causal agent [13]. In response, a conservation breeding program was established for *P. frosti* in 2010, with the initial aim of founding a viable assurance population for the species [1,14]. Having achieved this aim, the focus of the conservation breeding program has now shifted toward optimizing captive breeding and reintroduction protocols, in addition to safeguarding valuable genetic material from the species for future conservation applications. The aim of the present study was to apply reproductive technologies to this species for the first time in order to direct further applications. Specific objectives were twofold: (1) to determine the effect of hormone therapy (control, GnRHa, and GnRHa + MET) on spawning success in male–female pairs; and (2) to determine the effect of hormone therapy (control and hCG) on sperm-release and establish the peak time to collect sperm post-hormone administration in isolated males. Additionally, sperm suspensions collected during objective two were cryopreserved using established amphibian protocols and used to determine pre- and post-freeze sperm parameters. The remaining sperm samples were biobanked for long-term storage and future conservation applications.

## 2. Materials and Methods

### 2.1. Ethics Statement

A total of 40 male and 20 female Baw Baw frogs were employed for experimental purposes, as described herein. The protocols and procedures described were conducted following review and approval by the Zoos Victoria Animal Ethics Committee (ZV22009), in accordance with the National Health and Medical Research Council Australian code for the care and use of animals for scientific purposes.

### 2.2. Study Species

**Figure 1:**
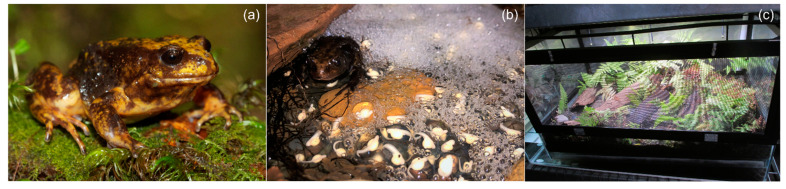
(**a**) Adult male Baw Baw frog, *Philoria frosti*. (**b**) Male Baw Baw frog shown with a foam nest and developing embryos. (**c**) Breeding enclosure with natural substrate and automated irrigation. Photographs courtesy of Damian Goodall, Zoos Victoria.

The Baw Baw frog is a medium-sized (svl; male ≈ 46 mm, female ≈ 55 mm) terrestrial species of the *Limnodynastidae* family [15] (Figure 1a). The species is restricted to a small area of protected montane gully habitat (1000–1300 m altitude) on the Mt Baw Baw Plateau in the Central Highlands of Victoria, Australia [14]. Commencement of the species’ breeding season corresponds with an increase in ambient temperature during austral spring [12]. Calling activity by male Baw Baw frogs occurs from September to March, with a peak in October and November which coincides with peak breeding activity [12]. Breeding occurs below ground, within natural cavities formed from vegetation, fallen logs, and rocks. These terrestrial nest-sites, while wet, typically retain little free water [12,14]. Amplexus is inguinal, and approximately 50–185 eggs are deposited into a foam nest [12] (Figure 1b). Multiple-male amplexus has been observed in captivity (D. Goodall, pers. obs.).

The species is listed as critically endangered by the Australian Department of Climate Change, Energy, the Environment and Water (DCCEEW) and the International Union for Conservation of Nature (IUCN).

### 2.3. Animal Husbandry

Baw Baw frogs were maintained in two identical isolated biosecurity facilities located at Zoos Victoria’s Melbourne Zoo (Parkville, VIC, Australia). Internal lighting within the facilities was controlled using a Photo Cell light-sensitive sensor set to replicate the local photoperiod. During the experimental period, the lighting was provided by LED plant spectrum tubes (Fluval) suspended above each shelf and programmed to provide a varying colour spectrum and varying intensities throughout the day. Ambient temperature within the facility was cycled annually to reflect seasonal changes in the average climatic conditions experienced in the alpine areas where the species naturally occurs. Annual temperatures range from 5 to 14 °C, including a 6-week overwintering period. Throughout the experimental period, frogs were maintained on an 8 °C/12 °C night/day temperature cycle.

Live feed was provided to the frogs twice weekly. Once per week, frogs were fed medium-sized crickets (*Acheta domestica*; ~3–5 crickets per individual) that were gut-loaded for ≥48 h prior to feeding with insect booster (Womberoo). Crickets were dusted with a multivitamin supplement (Multical dust, Vetafarm) before every feed. Every alternate feed, frogs were provided with pill bugs (*Armadillidium vulgare*; ~3–5 pill bugs per individual).

### 2.4. Experiment One: Hormone-Induced Spawning

To determine the effect of hormone therapy on spawning success, 20 male–female pairs were allocated to one of three experimental treatments (*n* = 6–7 pairs per treatment): 0 μg/g GnRHa (leuprorelin acetate; Lucrin; Provet, Erskine Park, Australia) (control group, *n* = 6 pairs), 0.5 μg/g GnRHa (*n* = 7 pairs), or 0.5 μg/g GnRHa + 10 μg/g metoclopramide (MET; Sigma-Aldrich, Macquarie Park, Australia) (*n* = 7 pairs). The formulation selected was based on previous research [16,17]. Individuals within each male–female pair were administered a single hormone dose corresponding to their experimental treatment. Males and females within each pair were injected at the same time prior to being placed in a breeding tank, as previous research has shown that this approach is more effective compared with administering hormones to male frogs 24- or 48 h prior to females [18]. Immediately prior to hormone injection, frogs were weighed to the nearest 0.01 g, and the dose administered was adjusted according to an individual’s body mass. Hormones were diluted in 100 μL of simplified amphibian ringer (SAR; composition (in mM): NaCl 113; KCl 2; CaCl_2_ 1.35; NaHCO_3_ 1.2) and administered via subcutaneous injection into the dorsal lymph sac, using ultra-fine 31-guage needles, following hormone-injection protocols used previously [19,20,21]. All frogs were sexually mature and ranged in weight from 9.1 g to 20.7 g (mean± SEM male mass (*n* = 20) = 11.56 ± 0.35 g; mean± SEM female mass (*n* = 20) = 16.66 ± 0.52 g). The body mass of males and females did not differ significantly between treatment groups (one-way analysis of variance (ANOVA), male mass: F_2,19_ = 0.067, *p* = 0.936; female mass: F_2,19_ = 0.707, *p* = 0.507).

Immediately following hormone injection, male–female pairs were placed into a breeding enclosure, one pair per enclosure. The breeding enclosures consisted of a glass aquarium with a raised ventilated canopy and automated irrigation connected to reverse osmosis (R.O.) water (Figure 1c). Each enclosure contained a layer of aquarium gravel, which varied in depth throughout the enclosure to create shallow pools; established live plants; and pieces of curved bark or plastic to offer a variety of oviposition sites within each enclosure (Figure 1c). Following hormone treatment, breeding enclosures were checked daily for the presence of eggs. Within a week of oviposition, the number of eggs deposited and embryonic development (fertilisation success) were scored. Unfertilised eggs or those exhibiting early embryonic failure were carefully removed from the clutch to avoid decomposing eggs negatively influencing viable embryos. Two weeks following hormone administration, if eggs were absent within the breeding enclosure, the male–female pair was categorised as unresponsive. Experiment One was conducted from 26 September to 10 October 2022, during the peak of the species’ natural breeding season.

### 2.5. Experiment Two: Hormone-Induced Spermiation and Sperm Cryopreservation

To determine the effect of hormone therapy on sperm-release and establish the peak time to collect sperm post-hormone administration, 20 male Baw Baw frogs were allocated to one of two experimental treatments: 0 IU/g hCG (purified human chorionic gonadotropin; Chorulon) (control group, *n* = 4) or 20 IU/g hCG (*n* = 16). The hormone hCG was selected, as previous research has reported that males from species within the *Limnodynastidae* family exhibit a more favourable sperm-release response to hCG compared to GnRHa [19]. Frogs received a single hormone dose, corresponding to their experimental treatment, diluted in 100 μL of SAR and injected subcutaneously into the dorsal lymph sac, using ultra-fine 31-gauge needles, following the hCG preparation and injection protocols used previously [20,22]. Prior to hormone injection, each frog was weighed to the nearest 0.01, g and the hormone dose administered was adjusted according to the individual male’s body mass. Males ranged in mass from 8.51 g to 15.32 g (*n* = 20, mean ± SEM = 10.70 ± 0.35 g). Immediately prior to the administration of hormones, a urine sample was collected from each male, and in all cases, the urine sample was aspermic (contained no sperm). Post-hormone injection, frogs were placed in individual 440 mL cylindrical plastic containers (11 cm D × 6 cm H), each with five Kimwipe tissues added, wetted with 25 mL of R.O. water. Hydrating individuals according to this procedure was essential in order to permit spermic urine collection at each of the sampling times (0, 2, 4, 6, 8, 10, and 12 h post-hormone injection). Urine volume at each collection time point ranged from 2 to 100 µL (*n* = 140, mean ± SEM = 33 ± 2.2 µL). Spermic urine was collected and assessed according to methods adapted from previous studies [20,21,22]. Urine samples were collected by gently inserting the tip of a 50 μL glass microcapillary tube, which was fire polished and cooled, into the opening of the cloaca until urination occurred. Spermic urine volume was measured, and the sample was immediately prepared for the assessment of total sperm, sperm concentration, and sperm viability. Note that sperm viability was the only measure of quality assessed for all spermic urine samples collected from each individual, at every time point. Sperm motility parameters were only quantified where spermic urine samples were above 50 μL in order to maximise the volume of spermic urine remaining to allow for sperm cryopreservation (see below). Sperm concentration (total sperm concentration/mL) was determined using an Improved Neubauer Haemocytometer (Bright Line, ProSciTech, Thuringowa, Australia). The total number of sperm released was then calculated by multiplying sperm concentration (sperm/μL) by spermic urine volume (μL). Sperm viability was quantified by mixing a 2–10 μL aliquot of spermic urine with 5 μL of a 1:50 dilution of SYBR-14, followed by 2 μL of propidium iodide (Invitrogen, L-7011, Thermo Fisher Scientific, Melbourne, Australia). The sample was incubated in the dark for 5 min following the addition of each solution. Wet mounts were prepared, and the proportion of viable sperm was evaluated under a fluorescent microscope. Sperm cells that fluoresced bright green were recorded as viable (live), while those that fluoresced red were recorded as non-viable (dead). Experiment Two was conducted from 28 to 29 September 2022, during the peak of the species’ natural breeding season.

#### Sperm Cryopreservation

The spermic urine samples remaining following the assessments described above were either cryopreserved individually (volume permitting) or pooled within an individual across two time-collection points. An aliquot of each pooled spermic urine sample was assessed for sperm concentration and viability according to the methods described above. Additionally, the sperm motility parameters (progressive sperm motility and total sperm motility) of raw spermic urine were assessed by placing a 2 µL aliquot of spermic urine under phase contrast (×400 magnification). A total of 100 sperm cells were visually assessed and categorized as progressively motile (forward movement regardless of direction), non-progressively motile (stationary with flagella beating), or immotile. Following assessment, 25–90 µL subsamples of spermic urine were slowly extended in concentrated cryoprotectant solution (20% (*w*/*v*) trehalose + 20% (*v*/*v*) dimethylformamide (DMF) in SAR, [23]) at a dilution of 1:1 (sperm: cryoprotectant solution), incubated at 4 °C for 10 min, and loaded in to 0.25 mL capacity straws (50–110 µL per straw). Samples were frozen using a field-friendly technique (dry shipper) adapted from a previous study [24] to provide a cooling rate of approximately 21 °C per minute. This was achieved by lowering the canister containing the goblet of straws (held on a cane) into a fully charged but empty dry shipper (Worthington Industries, Columbus, OH, USA, Model CX100 or CXR100) until the top rim of the canister was level with the top rim of the dry shipper; the canister was then held for 30 s before being lowered rapidly (1 s) to the bottom of the shipper. After ≥10 min in the dry shipper, the straws were plunged into liquid nitrogen. A small subset of samples were thawed (5 s in a water bath at 40 °C [23]) and activated by dilution in purified water at a ratio of 20:80 (sperm:water, *v*/*v*) to evaluate post-thaw sperm recovery. The remaining samples were stored in a dry shipper until accession into the biobank.

### 2.6. Statistical Analyses

In Experiment One, the number of male–female pairs ovipositing was compared between treatment groups by using two-tailed Fisher’s exact tests. ANOVAs were used to test for statistical differences in the mean number of eggs oviposited or percent fertilisation between experimental treatments. Comparisons among treatment means were conducted using Tukey–Kramer honest significant difference (HSD) post hoc tests. To verify homogeneity of variances, Levene’s tests were performed. In Experiment Two, the number of males releasing sperm was compared between treatment groups, using two-tailed Fisher’s exact tests. To assess the effect of sampling time on the quantity and quality of sperm released by males receiving hormone treatment, Linear Mixed Effects (LMEs) models fitted with restricted maximum likelihood (REML) were performed. Within each model, the sampling time was a fixed categorical effect; male ID was a random effect; and the response variable was either total sperm released, sperm concentration, or percent sperm viability. Prior to analysis, total sperm and sperm concentration data were log transformed using the transformation log_10_ (x + 0.01), and all percent sperm viability data were arc sine transformed using the transformation sin^−1^(√x). Body mass was not included in any of the LME models presented, as body mass was not significantly correlated with any of the response variables (total sperm, sperm motility, or sperm velocity, *p* > 0.05). All statistical analyses were performed using JMP Pro 16 software package (SAS Institute Inc., Cary, NC, USA). For all analyses, statistical significance was accepted at *p* < 0.05.

## 3. Results

### 3.1. Experiment One: Hormone-Induced Spawning

The number of male–female pairs ovipositing was highest in response to the administration of GnRHa combined with metoclopramide (71%), though, due to low sample size, the number of pairs ovipositing was not significantly different between treatment groups (Table 1; Fisher’s exact test, *p* > 0.05). Overall pairs that oviposited released between 40 and 80 eggs, with no significant differences in the number of eggs released or fertilisation success between treatment groups (Table 1).

### 3.2. Experiment Two: Hormone-Induced Spermiation and Sperm Cryopreservation

**Figure 2:**
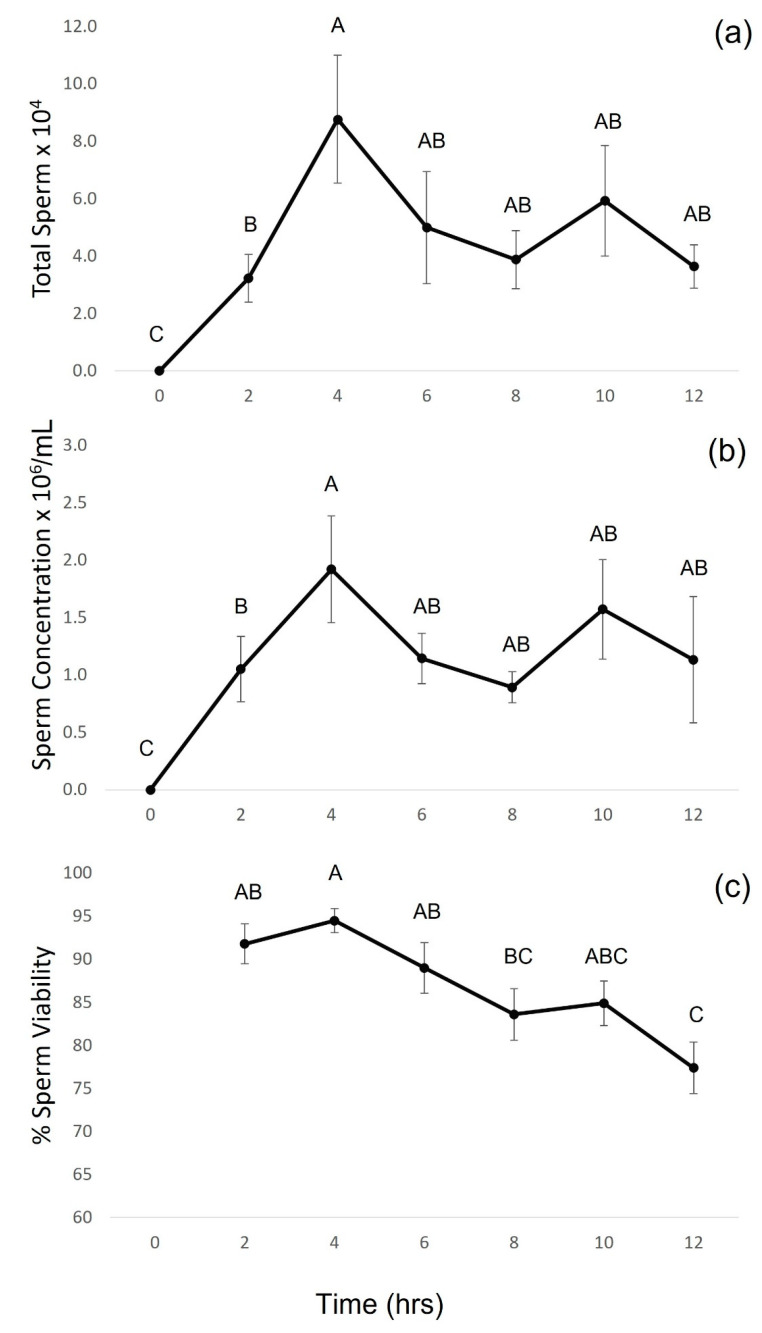
Effect of sampling time on (**a**) total number of sperm released (sperm × 10^4^), (**b**) concentration of sperm released (sperm × 10^6^/mL), and (**c**) percent sperm viability over a 12-h period post-administration of 20 IU/g hCG. Data are shown as the untransformed mean ± SEM (*n* = 16). Letters displayed are the result of Tukey–Kramer HSD post hoc tests on transformed data. Treatments that share a letter are not significantly different (*p* ≥ 0.05).

The number of males releasing sperm in response to 0 IU/g hCG (0%) was significantly lower than the number of frogs releasing sperm in response to the administration of 20 IU/g hCG (100%; Fisher’s exact test, *p* = 0.0002). For the males that received hormone administration (20 IU/g hCG, *n* = 16), the mean total sperm released and sperm concentration (sperm/mL) differed significantly over time (total sperm: LME; F_6,90_ = 107.10, *p* < 0.0001; sperm concentration: LME; F_6,90_ = 117.72, *p* < 0.0001). Both the total sperm and sperm concentration peaked at 4 h post-hormone administration, after which point both variables were lower though not significantly (Figure 2a,b). The percent sperm viability also differed significantly over time (LME; F_5,72_ = 5.79, *p* = 0.0002), with sperm viability peaking at 4 h post-administration (94.5%, Figure 2c). While sperm viability showed a significant decline over time, the viability remained relatively high throughout the collection period, with sperm exhibiting above 84% viability between 2 and 10 h post-hormone administration before falling to 77% at 12 h (Figure 2c). Overall, the spermic urine volume permitted sperm motility parameters to be assessed in 17 samples collected between 2 and 12 h post-hormone administration. The total percent motility ranged from 82.3 to 89.8% (mean ± SEM (*n* = 17) = 86.1 ± 1.8%), while the percent progressive motility ranged from 46.8 to 64.1% (mean ± SEM (*n* = 17) = 55.5 ± 4.1%).

Overall, 22 spermic urine samples from a total of 13 individual males were cryopreserved, with samples ranging in sperm concentration from 0.43 to 6.7 × 10^6^ sperm/mL. A subset of samples (*n* = 4) were thawed to evaluate post-thaw sperm recovery, while the remaining samples (*n* = 18) were transferred to Zoos Victoria’s Amphibian Biobank located within the Ian Potter Australian Wildlife Biobank at Museum Victoria for long-term storage. Table 2 shows the mean ± SEM sperm percent viability and total and progressive motility for fresh and frozen–thawed samples.

## 4. Discussion

The value of reproductive technologies has long been recognised by agriculture, aquaculture, and biomedicine. In recent decades, reproductive technologies have been applied to wildlife to assist with threatened species recovery, safeguard genetic diversity for future conservation actions, and help curb unprecedented global rates of species decline. The aim of the present study was to apply reproductive technologies, for the first time, to the critically endangered Baw Baw frog, *Philoria frosti*. Specifically, we determined the effect of hormone therapy (control, GnRHa, and GnRHa + MET) on spawning success in male–female pairs, as well as the effect of hormone therapy (control and hCG) on sperm-release in isolated males, and we quantified the peak time to collect sperm post-hormone administration. Additionally, sperm suspensions were cryopreserved using established protocols and used to determine pre- and post-freeze sperm parameters.

The results from the present study showed that hormone therapy increased the percentage of pairs ovipositing from 33% in the control group to 71% and 57% of pairs in the GnRHa + MET, and GnRHa treatments, respectively. The differences were not statistically significant due to high inter-treatment variation associated with low sample size (*n* = 6–7 per treatment), and additional experiments are consequently being planned to further investigate these patterns. Irrespective of this, the present study provides another example of the successful administration of a combination of gonadotropin-releasing hormone analogue with a dopamine antagonist at inducing spawning in male–female pairs or groups of amphibians [16,17,25]. Dopamine is believed to interfere with the neuroendocrine control of vertebrate reproduction and attenuate the action of natural GnRH-molecules [11]. As such, the co-injection of GnRHa with a dopamine-antagonist has been suggested as a means to enhance the stimulatory effects of GnRHa administration [11]. At present, the effectiveness of this protocol remains unequivocal. The spawning success of the northern leopard frog (*Lithobates pipiens*), the Panamanian golden frog (*Atelopus zeteki*), and the American bullfrog (*Lithobates catesbeianus*), was not enhanced by the combined administration of GnRHa and a dopamine antagonist, compared with the administration of GnRHa alone at optimal doses [26,27,28]. Investigating the benefits of the combined use of GnRHa with a dopamine antagonist at inducing spawning in a diversity of amphibian species remains an important avenue for future research.

The administration of GnRHa (with or without the combined administration of a dopamine antagonist) is generally regarded as more effective at eliciting a consistent and predictable gamete-release response across amphibian species compared to the administration of hCG. This is particularly true for inducing spawning in male–female pairs or groups of amphibians, as GnRHa acts at the level of the pituitary (hypothalamic approach) to trigger a complex cascade of physiological changes and a broader stimulation of reproductive events [2]. By contrast, hCG bypasses the pituitary gland and exerts an effect directly on the gonads (hypophyseal approach). The administration of hCG as a single hormone/dose (in the absence of other hormones including GnRHa) is more commonly used to induce gamete-release in isolated individuals, particularly male amphibians. The effectiveness of hCG has been shown to vary among amphibian families, with males from species within the *Limnodynastidae* family being previously reported to respond more favourably to hCG compared to GnRHa [20]. As a member of the *Limnodynastidae* family, male Baw Baw frogs in the present study were similarly found to respond favourably to the administration of hCG, as predicted.

When quantifying the sperm-release response of male amphibians to hormone therapy, it is important to determine the peak period for the collection of sperm [10]. Species-specific variation in the timing of sperm-release responses to hormone treatment have been observed and are thought to reflect interspecific differences in mating system structure, which may influence a species’ capacity for sperm production and its basal androgens [2]. Consequently, it is important to quantify the sperm-release response of males at regular intervals post-hormone administration on a species-specific basis. In the present study, both the quantity (total sperm and sperm concentration) and quality (sperm viability) of sperm peaked at four hours post-hCG administration. Interestingly, sperm parameters remained high in both quality and quantity throughout the collection period (up to 12 h post-hormone administration) in this species. Based on these results, sperm of high quality and quantity can be targeted for collection anywhere between 4 and 12 h post-administration in the Baw Baw frog. It is expected that these sperm parameters would decline between 12 and 24 h post-hormone administration, as has been seen in other species [22,29]. Continued research quantifying sperm parameters over a 24-h collection period will be necessary to determine the time at which sperm quality and quantity significantly decline.

Quantifying the sperm-release response of male amphibians over time is necessary so that targeted collection times can be established for a species to ensure sperm of the highest quantity and quality are collected for subsequent reproductive technologies, including artificial fertilisation and cryopreservation. This is particularly important when sperm are to be biobanked, as current sperm cryopreservation protocols typically result in a significant loss of viability and motility, which was also observed in the present study. Post-thaw sperm viability and total percent motility reported herein (59% and 17%, respectively) are within the very broad range of values previously reported for amphibians (sperm viability = 10–83%; total percent motility = 0–80%, across 50 species, as reviewed in [30]). The large variation among species in post-thaw sperm parameters reported may be the result of differences in the specific cryosuspension ingredients used, including permeating and non-permeating cryoprotectants and/or differences in cooling and thawing rates [30] and/or inherent species differences in initial sperm quality or cold tolerance. Varying these factors will be the focus of continued protocol refinement for the cryopreservation of sperm from our study species in an attempt to further improve post-thaw sperm parameters.

## 5. Conclusions

Reproductive technologies have enormous potential to contribute to the propagation, genetic management, and conservation of threatened amphibian species. In recognition of this potential, the present study applied hormone-induced spawning, hormone-induced sperm-release, and sperm cryopreservation protocols to the critically endangered Australian Baw Baw frog. Overall, hormone-induced spawning protocols successfully increased the percentage of pairs ovipositing, with 71% of pairs ovipositing in the GnRHa + MET treatment, compared with 33% of the control pairs. The hormonal induction of sperm-release was highly successful, with 100% of males administered 20 IU/g hCG releasing high-quantity and -quality sperm. Both sperm quantity (total sperm and sperm concentration) and sperm viability peaked at 4 h post-hormone administration but remained at high-enough levels suitable for targeted collection up to 12 h post-administration. Finally, cryopreservation protocols were also successfully applied to our study species, with post-thaw parameters within the broad range previously reported for amphibians. The results reported herein will inform the further refinement of reproductive technologies for this species and other threatened amphibian species globally.

## Figures and Tables

**Table 1:** The effect of hormone treatment on spawning success.

Response Variable	Hormone Treatment			*p*-Value
	0 µg/g GnRHa (Control)	0.5 µg/g GnRHa	0.5 µg/g GnRHa + 10 µg/g MET	
Pairs ovipositing (%)	2/6 (33%) ^A^	4/7 (57%) ^A^	5/7 (71%) ^A^	
Total number of eggs	50 ± 0 ^A^	55 ± 8.6 ^A^	46 ± 2.5 ^A^	0.522
Percent fertilisation	45 ± 45 ^A^	36 ± 13.9 ^A^	48 ± 13.8 ^A^	0.876

Data shown are the number of pairs ovipositing/total number of pairs (pairs ovipositing) or mean ± SEM (total eggs, percent fertilisation) (*n* = 6–7 per treatment). Data were analysed using Fisher’s exact tests (pairs ovipositing) or one-way ANOVAs (total eggs, percent fertilisation). Letters displayed are the result of post hoc tests. Within a row, treatments that share a letter are not significantly different (*p* > 0.05). See Methods for details of all statistical analyses.

**Table 2:** Sperm parameters of fresh and frozen–thawed spermic urine samples.

	Fresh	Post-Thaw
Sample size	22	4
Percent viability (live/dead)	89.8 ± 1.5	58.7 ± 6.6
Percent total motility	84.2 ± 1.8	17.4 ± 5.4
Percent progressive motility	53.5 ± 3.3	2.4 ± 0.6

## Data Availability

Data requests can be directed to the corresponding author.
